# Reabilitação Cardíaca na Pessoa Transplantada com Distrofia Muscular de Emery-Dreifuss

**DOI:** 10.36660/abc.20220560

**Published:** 2023-06-29

**Authors:** Maria Loureiro, Carlos Branco, João Duarte, Gonçalo Coutinho, Maria Manuela Martins, André Novo

**Affiliations:** 1 Instituto Ciências Biomédicas Abel Salazar Porto Portugal Instituto Ciências Biomédicas Abel Salazar, Porto – Portugal; 2 Centro Hospitalar e Universitário de Coimbra Coimbra Portugal Centro Hospitalar e Universitário de Coimbra, Coimbra – Portugal; 3 Cintesis@RISE-NursID Porto Portugal Cintesis@RISE-NursID, Porto – Portugal; 4 Faculdade de Medicina de Coimbra Coimbra Portugal Faculdade de Medicina de Coimbra, Coimbra – Portugal; 5 Escola Superior de Enfermagem do Porto Porto Portugal Escola Superior de Enfermagem do Porto, Porto – Portugal; 6 Instituto Politécnico de Bragança Bragança Portugal Instituto Politécnico de Bragança, Bragança – Portugal

**Keywords:** Transplante Cardíaco, Distrofia Muscular de Emery-Dreifuss, Reabilitação Cardíaca

## Abstract

A distrofia muscular de Emery-Dreifuss é uma doença neuromuscular hereditária rara. Suas manifestações começam principalmente na infância. As manifestações mais frequentes são fraqueza muscular progressiva, atrofia que geralmente se inicia na região escápulo-vertebral, estendendo-se posteriormente para a cintura pélvica e rigidez da coluna vertebral. Os pacientes também podem manifestar envolvimento cardíaco como palpitações, síncope, intolerância ao exercício, insuficiência cardíaca congestiva e distúrbios variáveis do ritmo cardíaco. ^1 - 3^ A presença e a gravidade dessas manifestações podem variar de acordo com o indivíduo e os subtipos da doença. ^2^ O envolvimento cardíaco é a característica mais preocupante desta doença, havendo alguns relatos da necessidade de transplante cardíaco nesta distrofia. ^4^

## Introdução

Um programa de reabilitação para incapacidade funcional por distrofia tem sido considerado uma opção terapêutica.^5 - 11^ No caso da pessoa com transplante cardíaco (TC), recomenda-se a reabilitação cardíaca (RC), centrada na componente de exercício físico. Um programa de RC após TC pode retardar complicações, manter a capacidade funcional e melhorar a qualidade de vida, o que é vital nesses pacientes.^6 , 7^ As recomendações atuais mostram que a RC reduz a mortalidade cardiovascular e as hospitalizações, melhorando a capacidade funcional e a qualidade de vida percebida.^12^

Os programas de RC são geralmente divididos em três fases. Fase I, que ocorre durante o período de internação e consiste, progressivamente, em exercícios respiratórios, mobilizações polissegmentais, deambulação, autonomia nas atividades de vida diária e início do programa educativo adequado (informação/ensino sobre a doença, tratamento/medicação, alimentação, exercício físico e controle de fatores de risco cardiovascular). A fase II ocorre após a alta, em ambiente ambulatorial, onde a pessoa frequenta um programa de exercícios personalizado e supervisionado dando continuidade ao programa educacional iniciado anteriormente. A fase III é chamada de manutenção, na qual o objetivo principal é acompanhar e monitorar a pessoa, conduzindo-a a um estilo de vida mais saudável e à adesão e manejo adequados do regime terapêutico.^13^

Os autores descrevem o caso de um homem de 32 anos com insuficiência cardíaca (IC) avançada secundária a distrofia muscular de Emery-Dreifuss (EDMD) que foi submetido à TC e foi incluído em um programa de RC.

### Caso Clínico

Homem de 32 anos diagnosticado com EDMD aos 12 anos de idade. Era portador heterozigoto da variante LMNA c.136A>G p.(Ile46Val), apresentando algum fenótipo característico dessa variante do gene: fraqueza muscular, contraturas e comprometimento cardíaco. Ele foi submetido a uma cirurgia no tendão de Aquiles e na articulação do cotovelo aos 15 anos por causa da atrofia muscular nos membros superiores e inferiores, com diminuição da amplitude de movimento articular.

Em 2018 foi identificado acometimento cardíaco decorrente da distrofia, com flutter atrial e trombo no apêndice atrial esquerdo, tendo feito implante de CRT-D e desde então hipocoagulado com varfarina. Nega história familiar de doenças cardiovasculares relevantes ou morte súbita, tem tabagismo ativo como fator de risco cardiovascular associado e IMC normal – 20,1.

Até 2020, o paciente foi internado várias vezes com IC, desenvolvendo cardiomiopatia dilatada não isquêmica (classe IV-NYHA) que não respondeu à terapia padrão para IC. Antes do transplante, apresentava perda funcional contínua associada à intolerância à atividade, tendo abandonado o exercício físico e as competições federadas de tênis de mesa desde 2018. O paciente foi transferido para nosso centro de TC ortotópico (INTERMACS 4), com menos de 24 horas entre o encaminhamento e transplante. Quando ele veio, seu nível de creatina quinase (CK) estava elevado (950 UI/l), e um estudo neurológico identificou hiporreflexia miotática, atrofia moderada, leve fraqueza muscular de membros superiores, extensão do antebraço limitada a +120/130°, contraturas do tendão de Aquiles com limitação da flexão dos pés e mobilidade da coluna vertebral preservada.

Segundo ele, nunca havia sofrido nenhuma intervenção de reabilitação, por isso foi integrado ao programa de RC pós-transplante ( Tabela 1 ). Para definir o programa de RC, foram consideradas as limitações da distrofia e o fenótipo além das condições pós-transplante. Apenas as limitações articulares foram condicionantes ao longo dos meses de intervenção, não havendo eventos adversos associados.

**Tabela 1:** – Plano Individualizado de Reabilitação Cardíaca - 3 fases

Fase I Hospitalização (11 dias)	Fase II (Até 2 meses)	Fase III (6 meses)
**– Exercícios de respiração** – Treino de Força - 0,5-1kg – Treino aeróbico (esteira, caminhada, bicicleta estacionária) – Alongamentos (limitado às amplitudes articulares) **15-30 minutos duas vezes ao dia/pós-extubação (PO1)**	– Exercícios de respiração aquecimentos/alongamentos – Treinamento aeróbico- (escadas, caminhada, bicicleta ergométrica) – Treinamento de força -1,5-2kg (limitações de esternotomia) **180 minutos/semana**	– Treino aeróbico - (escadas, caminhada, bicicleta estacionária) – Treinamento de força - 3-5kg- 30 minutos- 3 vezes/semana – Treino de tênis de mesa – 60 minutos,2 vezes/semana **210 minutos/semana**
Instruído sobre sinais e sintomas de alarme (FC máxima não superior a 20bpm da FC inicial, Borg modificado ≤5, tontura, dor no peito, sudorese moderada). Ensino sobre estilos de vida saudáveis (dieta, medicação, fatores de risco cardiovascular).	Instrução sobre sinais e sintomas de alarme. Ensino sobre estilos de vida saudáveis.	Continuidade do estilo de vida saudável e cumprimento das orientações de saúde monitoradas.

FC: frequência cardíaca.

## Discussão

São apresentados os domínios avaliados durante o programa de RC e suas implicações ( Tabela 2 ). Com relação ao uso de exercícios respiratórios, foi possível mensurar o aumento do volume de reserva inspiratória ao longo da intervenção. Embora ainda não tenha atingido valores considerados para a população portuguesa saudável, pode-se inferir que houve melhora clinicamente significativa da capacidade funcional por meio da avaliação do teste de caminhada de 6 minutos. Em relação à força, e apesar de se tratar de uma pessoa com déficit na amplitude de movimento articular, foi possível o aumento da força medida pela dinamometria. Todos esses ganhos promoveram uma melhora na capacidade de autocuidado e no desenvolvimento das atividades diárias, o que influencia na qualidade de vida percebida ( Tabela 3 ).

**Tabela 2:** – Resultados monitorados ao longo das 3 fases do programa de reabilitação cardíaca

Resultados		Fase I	Fase II	Fase III
**Inspirometro de incentivo volumétrico (max.4000ml)**		1900ml	2900ml	3200ml
**Teste de caminhada de 6 minutos**	**Esperado** Equação de Enright e Sherril	718	714,48	710,08

	**Alcançado**	**310**	**400**	**510**
**Dinamômetro Dinamômetro aneroide-Mão dominante (Kgf)**		15	23	40
**Fração de ejeção (ecocardiograma transtorácico)**		70%	67%	65%

**Tabela 3:** – Avaliação da Qualidade de Vida (EuroQol 5D5L)

Estágio	Mobilidade	Autocuidados	Atividade	Dor/Desconforto	Ansiedade depressão	EQ-5D-5L Código	Índice EQ-5D-5L
I	3	3	4	4	4	33444	0,133
II	2	2	2	2	3	22223	0,587
III	2	1	1	1	4	21114	0,644

Assim, a qualidade de vida melhorou, com evolução mais significativa nos itens Autocuidado, Atividade e Dor/Desconforto. De referir que o domínio Ansiedade/Depressão volta a agravar-se na fase 3 (com menor seguimento).

## Conclusão

Este relatório aponta para a importância da integração dos pacientes transplantados cardíacos no programa de RC. Mesmo com limitações prévias, é possível melhorar a capacidade funcional, volume de reserva inspiratória, força e qualidade de vida em pacientes com EDMD.
