# Supplementary material for: Reabilitação Cardíaca na Pessoa Transplantada com Distrofia Muscular de Emery-Dreifuss
Source: Arq Bras Cardiol. 2023 Jun 29;120(7):e20220560. [Article in Portuguese] doi: 10.36660/abc.20220560 (PMC10364993; doi:10.36660/abc.20220560)
Supplement: Translation

# Translation: Cardiac Rehabilitation in a Transplanted Person with Emery-Dreifuss Muscular Dystrophy

**Authors:** Maria Loureiro, Carlos Branco, João Duarte, Gonçalo Coutinho, Maria Manuela Martins, André Novo

**Affiliations:** 1 Portugal Instituto Ciências Biomédicas Abel Salazar, Porto – Portugal; 2 Portugal Centro Hospitalar e Universitário de Coimbra, Coimbra – Portugal; 3 Portugal Cintesis@RISE-NursID, Porto – Portugal; 4 Portugal Faculdade de Medicina de Coimbra, Coimbra – Portugal; 5 Portugal Escola Superior de Enfermagem do Porto, Porto – Portugal; 6 Portugal Instituto Politécnico de Bragança, Bragança – Portugal

**Keywords:** Heart Transplant, Emery-Dreifuss Muscular Dystrophy, Cardiac Rehabilitation

## Abstract

Emery-Dreifuss muscular dystrophy is a rare hereditary neuromuscular disease. Its manifestations begin primarily in childhood. The most frequent manifestations are progressive muscle weakness, atrophy that usually begins in the scapula-vertebral region, extending later to the pelvic girdle, and spinal stiffness. Patients can also manifest cardiac involvement as palpitations, syncope, exercise intolerance, congestive heart failure, and variable heart rhythm disturbances.^1 - 3^The presence and severity of these manifestations can vary according to the individual and the disease’s subtypes. ^2^ Cardiac involvement is the most worrisome feature of this disease, and there are some reports of the need for heart transplantation in this dystrophy. ^4^

## Introduction

A rehabilitation program for functional disability due to dystrophy has been considered a therapeutic option.^5 - 11^In the case of the heart-transplanted person, cardiac rehabilitation (CR) is recommended, focusing on the physical exercise component. A CR program after heart transplantation (HT) can delay complications, maintain functional capacity, and improve quality of life, which is vital in these patients.^6 , 7^Current recommendations show that CR reduces cardiovascular mortality and hospitalizations, improving functional capacity and perceived quality of life.^12^

CR programs are usually divided into three phases. Phase I, which occurs during the hospitalization period and consists, progressively, of respiratory exercises, polyssegmental mobilizations, walking, autonomy in daily life activities, and the beginning of the appropriate educational program (information/teaching about the disease, treatment/medication, diet, physical exercise, and control of cardiovascular risk factors). Phase II occurs after discharge, in an outpatient setting, where the person attends a personalized and supervised exercise program continuing the educational program previously started. Phase III is called maintenance, in which the main objective is to follow and monitor the person, leading him/her towards a healthier lifestyle and adequate adherence to and management of the therapeutic regimen.^13^

The authors describe the case of a 32-year-old man with advanced end-stage heart failure (HF) secondary to Emery-Dreifuss muscular dystrophy (EDMD) who underwent HT and was included in a CR program.

### Clinical case

32-year-old male diagnosed with EDMD at the age of 12 years. He was a heterozygous carrier of the variant LMNA c.136A>G p.(Ile46Val), presenting some characteristic phenotype of this gene variant: muscle weakness, contractures, and cardiac impairment. He underwent surgery on his Achilles tendon and elbow joint at age 15 because of muscle atrophy in the upper and lower limbs, with decreased joint range of motion.

In 2018 cardiac involvement arising from the dystrophy was identified, with atrial flutter and thrombus in the left atrial appendage, having undergone CRT-D implantation and since hypo coagulated with warfarin. He denies a family history of relevant cardiovascular diseases or sudden death, has active smoking as an associated cardiovascular risk factor, and has a normal BMI – of 20.1.

Up to 2020, the patient was hospitalized several times for HF, developing non-ischemic dilated cardiomyopathy (class IV-NYHA) that did not respond to the standard HF therapy. Before the transplant, he had continued functional loss associated with activity intolerance, having given up physical exercise and federated table tennis competitions since 2018. The patient was transferred to our center for orthotopic HT (INTERMACS 4), with less than 24 hours between referral and transplantation. When he came, his creatine kinase (CK) level was elevated (950 UI/l), and a neurological study identified myotatic hyporeflexia, moderate atrophy, mild upper limb muscle weakness, forearm extension limited to +120/130°, Achilles tendon contractures with limitation of feet flexion and preserved spinal column mobility.

According to him, he had never undergone any rehabilitation intervention, so he was integrated into the post-transplant CR program ( Table 1 ). To define the cardiac rehabilitation program, dystrophy limitations, and phenotype were considered beyond post-transplant conditions. Only the joint limitations were conditioning factors throughout the months of intervention, and there were no associated adverse events.

**Table 1:** – Individualized Cardiac Rehabilitation Plan - 3 phases

Phase I Hospitalization (11 days)	Phase II (Up to 2 months)	Phase III (6 months)
**– Breathing exercises** – Strength Training - 0,5-1kg – Aerobic training (treadmill, walking, stationary bike) – Alongamentos (limitado às amplitudes articulares) **15-30 minutes twice a day/post-extubation (PO1)**	– Breathing exercises Warm-ups/stretches – Aerobic training- (stairs, walking, stationary bike) – Strength training -1.5-2kg (sternotomy limitations) **180minutes/week**	– Aerobic training - (stairs, walking, stationary bike) – Strength training - 3-5kg- 30 minutes- 3 times/week – Table tennis training – 60 minutes, 2 times/week **210 minutes/week**
Instructed on alarm signs and symptoms (maximum HR not to be more than 20bpm of initial HR, modified Borg ≤5, dizziness, chest pain, moderate sweating). Teaching about healthy lifestyles (diet, medication, cardiovascular risk factors).	Instruction about alarm signs and symptoms. Teaching about healthy lifestyles.	Continuity of healthy lifestyle and compliance with health guidelines monitored.

HR: heart rate.

## Discussion

The domains evaluated during the CR program and the implications are presented ( Table 2 ). Regarding the use of breathing exercises, measuring an increase in the inspiratory reserve volume throughout the intervention was possible. Although it still did not reach values considered for the healthy Portuguese population, it can be inferred that there was a clinically significant improvement in functional capacity by assessing the 6-minute walk test. Regarding strength, and despite dealing with a person with a deficit in joint range of motion, an increase in strength was possible, as measured by dynamometry. All these gains promoted an improvement in self-care capacity and the development of daily activities, which influences the perceived quality of life ( Table 3 )

**Table 2:** – Results monitored throughout the 3 phases of the cardiac rehabilitation program

Results		Phase I	Phase II	Phase III
**Volumetric incentive inspirometer (max.4000ml)**		1900ml	2900ml	3200ml
6-minute walk test	**Expected** Enright & Sherril’s equation	718	714.48	710.08

	**Reached**	**310**	**400**	**510**
**Dynamometer aneroid dynamometer-Dominant hand (Kgf)**		15	23	40
**Ejection fraction (transthoracic echocardiogram)**		70%	67%	65%

**Table 3:** – Quality of Life Assessment (EuroQol 5D5L)

Phase	Mobility	Self-care	Activity	Pain/Discomfort	Anxiety/Depression	EQ-5D-5L Code	EQ-5D-5L Index
I	3	3	4	4	4	33444	0.133
II	2	2	2	2	3	22223	0.587
III	2	1	1	1	4	21114	0.644

Thus, quality of life was improved, with the most significant evolution in the items Self-Care, Activity, and Pain/Discomfort. It should be noted that the Anxiety/Depression domain worsens again in phase 3 (with less follow-up).

## Conclusion

This report points to the importance of integrating heart transplant patients into CR program. Even with previous limitations, improving functional capacity, inspiratory reserve volume, strength, and quality of life is possible in patients with EDMD.
